# English Criticisms of Americans

**Published:** 1882-12

**Authors:** 


### Editorial.

### ENGLISH CRITICISMS OF AMERICANS.

The English nation has adopted several different postures toward this country and its inhabitants, since the former ceased to be its most prosperous and promising colony, and the latter its most humble subjects. It began at an early period by waging actual warfare, whose net results to the mother country in the loss of pounds, shillings and pence it is impossible to compute. To this period of hostilities, there succeeded a series of years in which the United States and its people were either completely ignored in Great Britain, or regarded with a species of contempt which there was not even a pretense of concealing. The hideous caricatures of American life and manners drawn by the pencil of the late Mr. Chas. Dickens, of whom, by the way, it is said that he never drew the portrait of an English gentleman, were accepted as truthful by the mass of his trans-Atlantic readers. His example was followed by a host of less popular authors among his countrymen, who had either never visited this country or whose knowledge of it was limited to a few weeks' visit to the city of New York and a trip to Niagara Falls. His latest disciples in this species of elegant literature, are two English story-writers, named Besant and Rice, who seem to be engaged in spicing the same stale meats for the palate of their countrymen. They have recently portrayed, as a candidate for a seat in the British House of Peers, a filthy, ignorant and otherwise disgusting fictitious character, whose native place they fix in "Canaan City, New Hampshire." This spectacle of an American citizen with the dress and manners of a "yahoo" seems to have unfailing attractions for the British public.

But the latest posture of the refined and educated among the English people is, as regards this country and its inhabitants, one in which animosity, ridicule and contempt seem to have given place to criticism. Decidedly this is more gracious and promising. For the reasons which lie at the basis of the change, we have not to go far nor search deeply. We are growing rich and strong. Nowhere in the world are wealth and power more worshiped than in that very "tight little isle." As a result, in place of their shot and in lieu of their sneers, we are tendered—advice!

Among the more recent of the English critics who have visited these shores, and certainly among the most distinguished, is Mr. Herbert Spencer. Here is a man pre-eminently honored among his contemporaries on both sides of the water for his profound learning, his literary acquirements and his philosophical writings. The latter will remain a monument to his intellectual greatness when half the books written in the nineteenth century are quite buried in oblivion. Naturally we listen to what he says of us with great respect and attention. His words are reproduced in the daily journals; and we notice also that some of the medical periodicals have made extracts from his farewell address.

We confess that, as we weigh him carefully, we can do no more than set down the distinguished speaker in the long list of English critics of American life and manners who have spoken with the most imperfect knowledge of their theme. Mr. Spencer has made the conventional trip, and done the conventional thing. He too made his hasty visit to a few of the Eastern cities, visited Niagara Falls, and made the usual diversion from the platitudes of an after-dinner speech to tell us, just before re-crossing the Atlantic, how very fearful he was of our future! Were this merely a political future, the subject might be relegated to the political press; but it is our physical future which distresses Mr. Spencer. He describes "degenerative tendencies in American life;" "extreme persistent activity;" "early turning gray of the hair;" "nervous collapses;" "cripplings by overwork;" "serious physical mischief;" "damaged constitutions;" "injury to posterity," and a great deal more of the same sort.

Now all this is, in the main, true of the people whom Mr.

Spencer happened to meet here; but it is none the less true of the people whom we meet in all the busy centers of civilization, London, New York, Chicago, Paris, Philadelphia. These are the bitter fruits of intense competition, overcrowding and avarice; the stress and strain of the struggle for existence among a mighty multitude of eager men. If he meant it to be inferred that this was, on the whole, more conspicuous in America than in England, the distinguished speaker was certainly in error. For every one illustration of this "degenerative tendency" he can find in the United States, we will point him to ten in his own country. For every type of nervous collapse he can point out in this people, we can refer him to a dozen whose histories are recorded in the writings of his own countrymen. What he says is not new to any of us here It has been told us in fully as elegant English as Mr. Spencer's, by American physicians, more particularly by neurologists. The representatives of our profession in Great Britain have none the less preached the same story again and again to their own brethren on the other side of the Atlantic.

The picture, indeed, presented us by Mr. Herbert Spencer in his late attitude, is not without its ludicrous and even absurd aspect. Think of it! Here is a man telling us that we are working too hard, and as a consequence physically degenerating, while the country, from which he comes to say it, has in it more overworked children and women, to say nothing of men, than any other on the face of the earth; a country whose nobles are to-day assassinated by Irish serfs, in the sheer desperation induced by the prospect of starvation; a country in some of whose populous districts there is such squalor and destitution as have aroused, even in the last twelvemonth, the utmost pity and sympathy of our reading public; a country, the misery, wretchedness and poverty of whose laboring classes have been by none more eloquently and graphically described than by English authors themselves.

This is the land which sent over to us the dolorous "Song of the Shirt," and Mrs. Elizabeth Barrett Browning's pitiful wail, called the "Cry of the Children!" This is the England from which our philosopher travels to tell us that we are losing our hair and physically degenerating, because we are working too hard!

One year ago, two members of the British House of Commons passed through Chicago. They had been visiting every part of the United States, its wheat fields, its cities, its prairies, its deserts and its mountains. They came to study and to learn; and they did their work well. They came in order to advise others intelligently with reference to investments in American farm lands. While here, they, too, were invited to a banquet by a number of cultivated gentlemen in this city. One of these two members of the British Parliament had visited America twenty years before, and was enabled thus to compare its present with its former condition. In response to a toast, he said:

"A great deal has been foolishly spoken and written on the subject of physical degeneration among Americans. I do not believe in a word of it! I have seen your people in every part of your land, and have found them in no respect physically inferior to those of any part of Great Britain. How could it be otherwise? We are amazed at your growth, your immense resources, your material accumulation of everything that can conduce to man's physical welfare. We have compared the students in your colleges and universities with our own at Oxford and at Cambridge, and we find them equal in bodily vigor and powers of endurance. The improvement in your physical type during the last twenty years, has been one of the most conspicuous and interesting of the many facts we have noted since we have been here among you. I am looking at this moment into he faces of say, one hundred gentlemen of your great city. In physical vigor, in all the outward evidences of good health and maturity of bodily development, they are certainly the equal of the next hundred guests I shall meet at a dinner-table in Great Britain. No, gentlemen, it is not the physical, it is the mental, the moral, the political future of your country, which ought to awaken your anxieties—the struggle which you are yet to witness between capital and labor, the contest between, on the one hand, the brute forces which your material wealth has brought into existence; and, on the other, the conservative energies of law, economical industry, and intellect, whose ultimate supremacy you will have, at all hazards, to secure."

So it appears that even our English critics are not all of one way of thinking.

If there be any timorous Americans, physicians or laymen, who, listening to Mr. Herbert Spencer's remarks on the eve of his departure for Europe, were thereby inclined to be, with him, fearful for the physical future of our people, let them take heart of grace! What Mr. Spencer does not know about this country, would fill many more volumes than those which he has himself already written. His assumption that a few New York gentlemen, whose hair had become prematurely gray in their anxieties over the Stock Exchange, were the whole American people, is only one of many British blunders. What folly to presume that the average man will suffer physical degeneration, in a land which has steadily advanced in the task of feeding the other nations of the world! Fortunately the great mass of the citizens of the United States, live outside the large cities. They are surrounded by unparalleled resources for physical development, and are profiting thereby to the largest extent. Their markets are unrivalled; their food, more wholesome, more abundant and cheaper than that of any other nation under the face of heaven. They are better clothed, day and night; tread on more carpets; sleep on more comfortable beds; warm themselves by more ample artificial heat-sources; and have more leisure, as the result of employing labor-saving machinery, than the people in any civilized or uncivilized race on the earth. They could have shown Mr. Spencer a locomotive engine, flying as straight as the crow flies, through consecutive hundreds of miles of waving corn, each stalk bearing eight full ears, and none measuring less than twelve feet in height. They could have pointed out to him their cattle upon a thousand hills, of which, after eating generously themselves, they will sell to his countrymen enough to make life a trifle less burdensome to the British laborer. Right here, in the State of Illinois, they could have introduced him last week to a "Banquet of Behemoths," where every guest weighed on the scales more than two hundred and sixty pounds before taking his seat at the groaning board.

We have not the slightest fear for the physical future of the average American; and we trust that Mr. Spencer and his fellow-critics may not be overburdened with their anxieties on this score. Nor have we space in which to mention by name the well-known gentlemen in public life who were or are superb examples of what this country has developed as types of manly excellence. The late President Garfield was a noble example of physical vigor; and his successor in office is certainly not lacking in avoirdupois. In the ranks of the United States Army, we remember the form of the gallant and lamented Custer, and the figures of Hancock and Augur; of the Hon. David Davis, of Illinois, and of the Hon. James G. Blaine, of Maine, in the the senate; the grand figure of the late chief-justice of the United States; and hosts of others in all the professions and occupations of life.

In our wars with the English people, we were not destroyed. Responding, later, to their contempt and sneers, we taught them telegraphy, and the use of the cylinder press and of a thousand other inventions which, by saving labor, have given them and us the greater leisure for physical development and culture. Let it be set down as a fact, that we shall not only survive their criticisms, but also disappoint their fears. Mr. Spencer complains that we do not grumble enough; and we shall establish the truth of this criticism at least, by refusing to grumble at him or his countrymen for their behavior, past and present toward us. We are great enough and strong enough to respond by simply laughing good-naturedly at him and them.

